# Supplementary material for: Proximity Mapping of CCP6 Reveals Its Association with Centrosome Organization and Cilium Assembly
Source: Int J Mol Sci. 2023 Jan 9;24(2):1273. doi: 10.3390/ijms24021273 (PMC9867282; doi:10.3390/ijms24021273)
Supplement: Supplementary file 1 [file ijms-24-01273-s001.zip › Supplementary Figures.pdf]

Supplementary Information

Proximity mapping of CCP6 reveals its association with centrosome organization and cilium assembly.

Sergi Rodriguez-Calado [1], Petra Van Damme [2], Francesc Xavier Avilés [1], Ana Paula Candiota [3,1], Sebastian Tanco [1,*] and Julia Lorenzo [1,*]

1 Institut de Biotecnologia i Biomedicina, Departament de Bioquímica i Biologia Molecular, Universitat Autònoma de Barcelona, 08193 Cerdanyola del Vallès, Barcelona, Spain

2 iRIP Unit, Laboratory of Microbiology, Department of Biochemistry and Microbiology, Ghent University, K. L. Ledeganckstraat 35, 9000 Ghent, Belgium

3 Centro de Investigación Biomédica en Red en Bioingeniería, Biomateriales y Nanomedicina (CIBER-BBN), 08193 Cerdanyola del Vallès, Barcelona, Spain

* Correspondence: julia.lorenzo@uab.cat (J.L.); sebastianmartin.tanco@uab.cat (S.T.). Tel.: +34 93 586 8957 (J.L.); +34 93 586 8938 (S.T.)

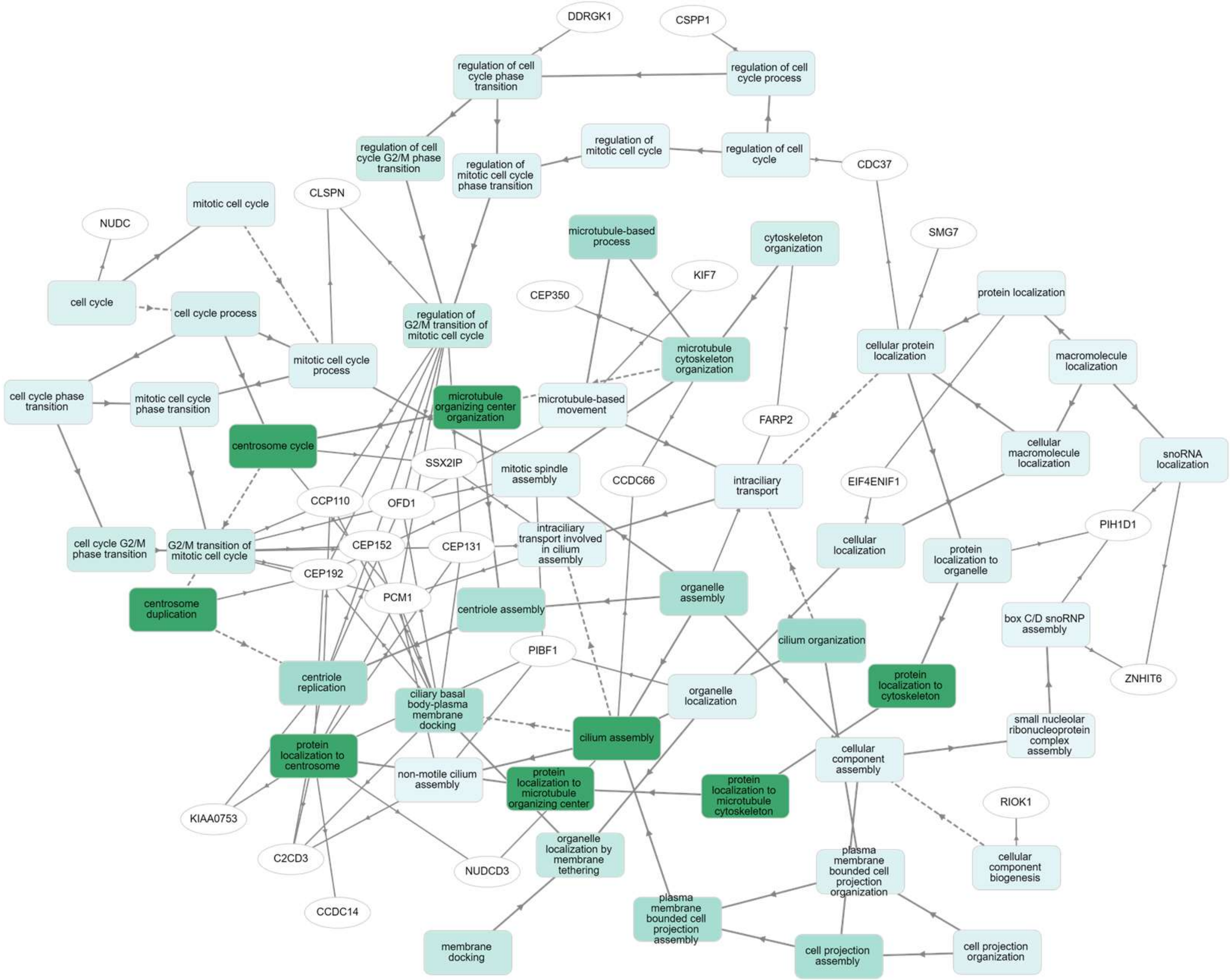

Figure S1. Network analysis of CCP6 proxeome in HEK 293 cells. CCP6 enriched (p-value < 0.05) proteins were analyzed using GOnet software. The figure shows GO terms and genes connected by two types of edges (connecting two GO terms or connecting a GO term and a gene). When the edge connects two GO terms the arrow is directed from the less specific term to the more specific term, whereas when the edge connects a GO term and a gene, it is always directed toward the gene. Dotted arrows indicate that a GO term is part of another one. GO term nodes are colored by the p-value of enrichment, being a more intense node coloring indicative of a higher significance.

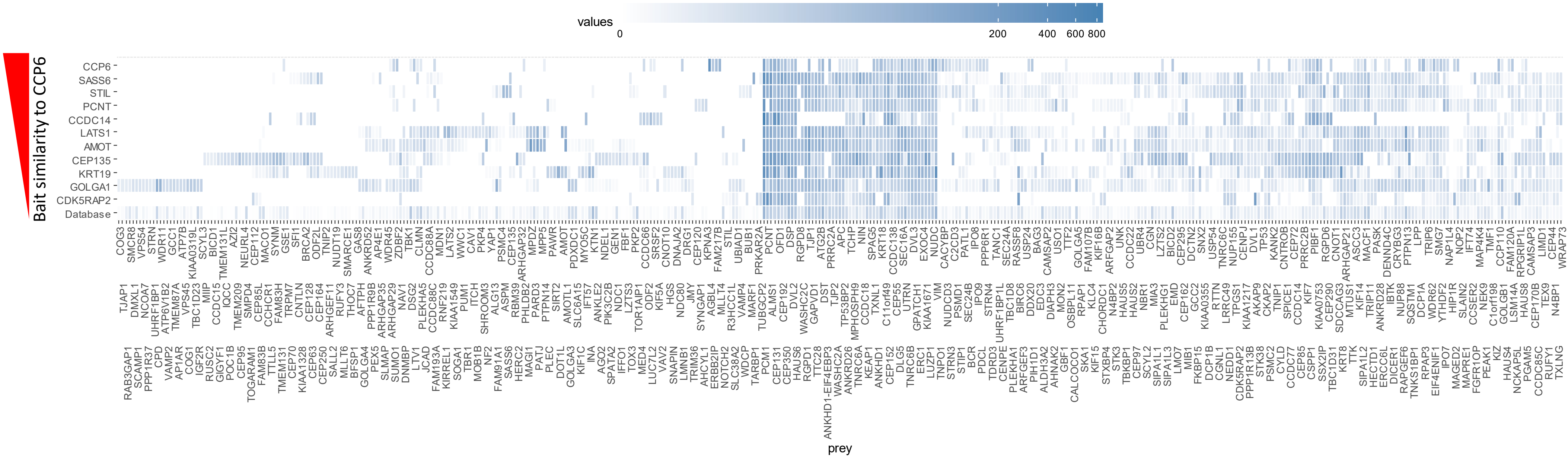

Figure S2. Heatmap representation of the average MS1 intensity as and abundance measure of the different preys in the BioID setups for CCP6 and the 10 most similar baits found in the entire Human Cell Map database. From top to bottom, baits are sorted from most similar to least similar to CCP6 as calculated by the Jaccard distance. Jaccard distances are as follows: 0.832 for SASS6, 0.843 for STIL, 0.846 for PCNT, 0.851 for CCDC14, 0.856 for LATS1, 0.860 for AMOT, 0.862 for CEP135, 0.868 for KRT19, 0.874 for GOLGA1 and 0.875 for CDK5RAP2. The Database row represents the average MS1 intensity for each prey, averaged across all baits in the Human Cell Map. For better visualization, intensity values were pseudo-log transformed.

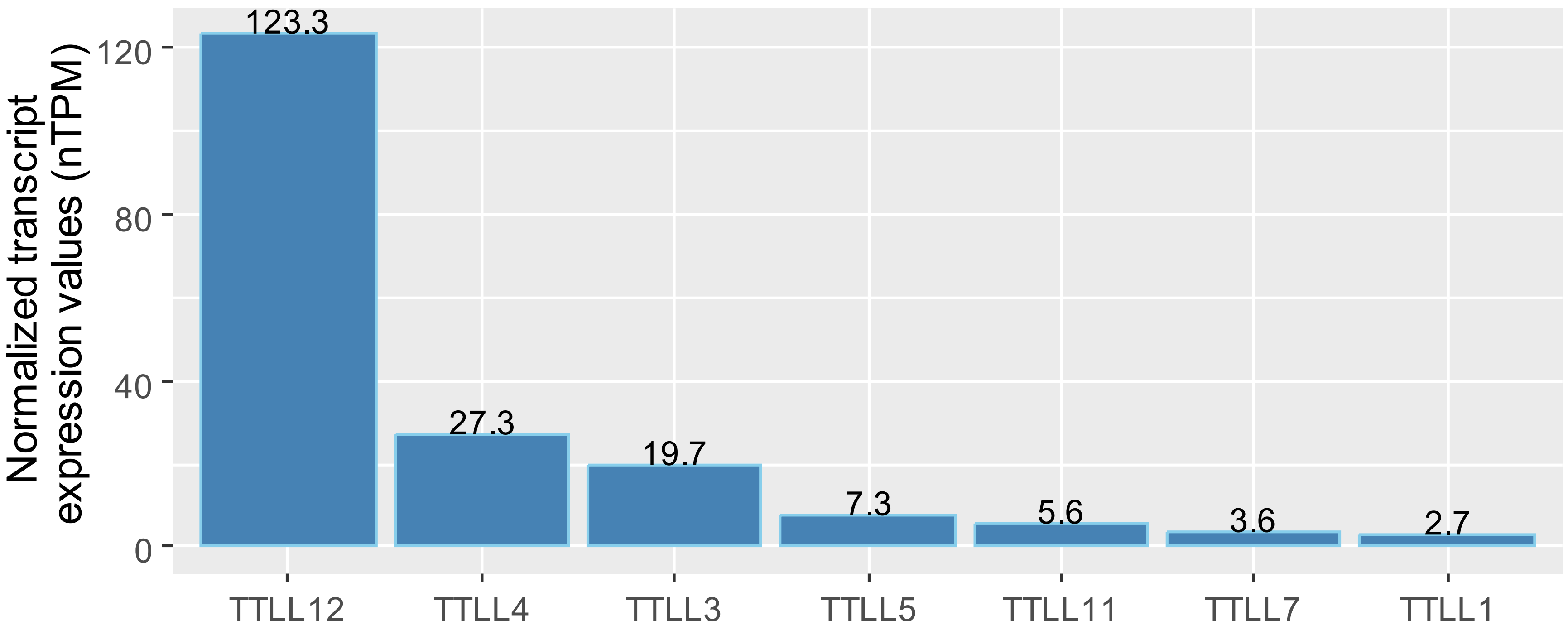

Figure S3. Normalized transcript expression levels for the different TTLLs in HEK 293 cells according to the Human Protein Atlas (https://www.proteinatlas.org/).

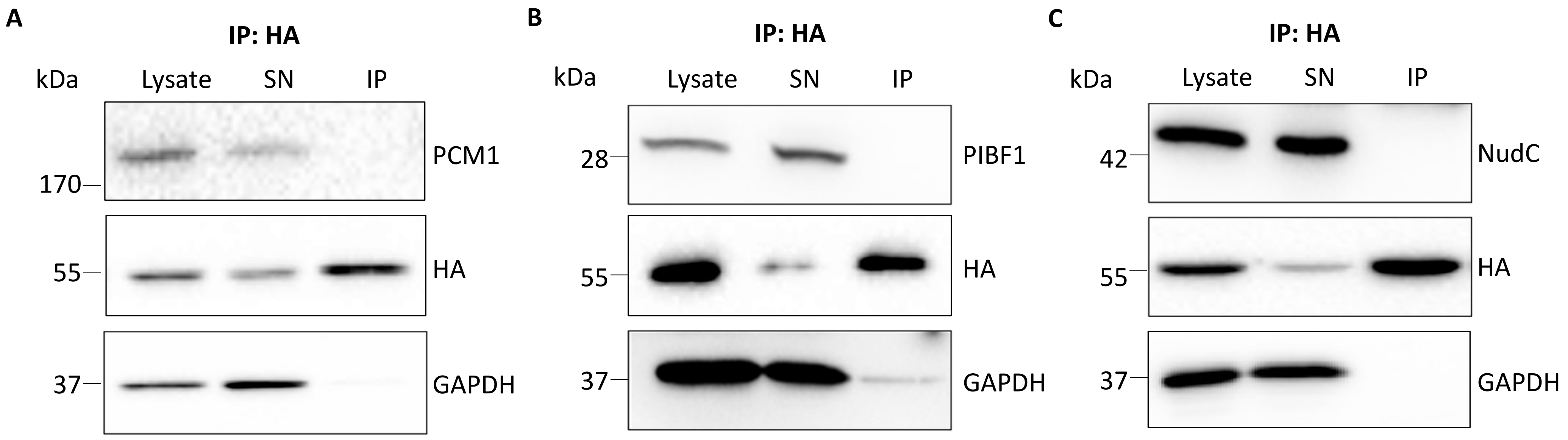

Figure S4. Characterization of the interaction between CCP6 and candidate interactors by co-immunoprecipitation. HA-tagged CCP6 was overexpressed in HEK293T cells and immunoprecipitated with a specific anti-HA antibody. The resulting supernatant (SN) and immunoprecipitate (IP) were blotted with specific antibodies for (A) PCM1, (B) PIBF1, and (C) NudC to detect the physical interaction. GAPDH was evaluated as a negative control of the immunoprecipitation procedure.

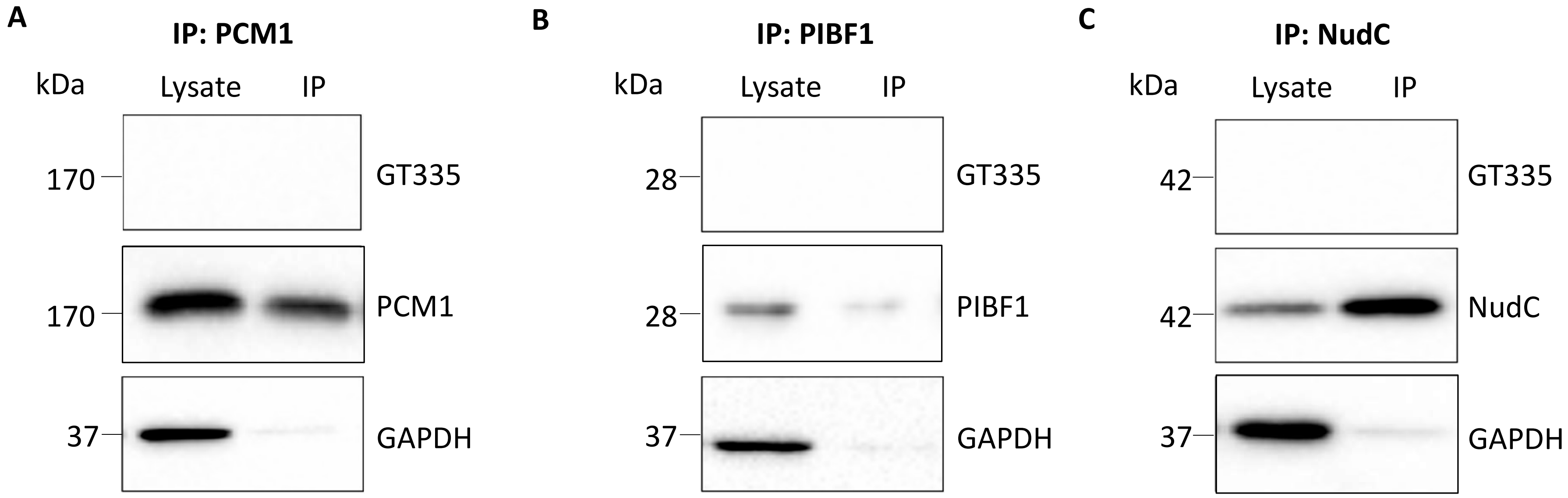

Figure S5. Characterization of the polyglutamylated state of (A) PCM1, (B) PIBF1 and (C) NudC. Proteins were pulled-down using specific antibodies and blotted with the GT335 antibody. GAPDH was evaluated as a negative control of the immunoprecipitation procedure.
